# Efficacy and safety of topical application of tranexamic acid in patients undergoing reconstructive plastic surgery after excision of facial skin cancers: a randomised clinical trial

**DOI:** 10.1590/0100-6991e-20243761-en

**Published:** 2024-07-05

**Authors:** VICTOR FELIPE DOS SANTOS TEJADA, LINJIE ZHANG, LUCIANO ZOGBI

**Affiliations:** 1 - Universidade Federal do Rio Grande - Rio Grande - RS - Brasil

**Keywords:** Tranexamic acid, Efficacy, Plastic Surgery Procedures, Ácido Tranexâmico, Procedimentos de Cirurgia Plástica, Eficácia

## Abstract

**Introduction:**

Tranexamic acid (TA) has attracted increased attention among surgical specialties, but its use in plastic surgery is limited. The aim of this study was to assess the efficacy and safety of topical administration of 3% TA solution in reconstructive surgery of the face and scalp after excision of skin cancers.

**Methods:**

a randomized, double-blind, parallel-group clinical trial was conducted in patients aged 18 years or older with malignant skin neoplasms in the face or scalp region (ICD-10 C44.9). The primary outcome was volume of blood loss in the intraoperative and immediate postoperative period. Secondary outcomes included difficult-to-control intraoperative haemorrhage, hematoma, ecchymosis, and other adverse events.

**Results:**

of the 54 included patients, 26 were randomised to TA group and 28 to placebo group. The mean blood loss was 11.42ml (SD 6.40, range 8.83-14.01) in the TA group, and 17.6ml (SD 6.22, range 15.19-20.01) in the placebo group, representing a mean decrease of 6.18ml (35.11%) (p=0.001). TA significantly reduced the risk of ecchymosis (RR = 0.046; 95% CI: 0.007-0.323). Only two patients in the placebo group experienced ischemia in the flaps, and one patient in the placebo group experienced tissue necrosis requiring surgical reintervention. There were no surgical wound infections, thromboembolic phenomena, or other adverse events related to TA.

**Conclusions:**

topical TA may reduce intraoperative and immediate postoperative bleeding, with a significantly decreased risk of ecchymosis. There is no evidence of ischemic damage of flaps, systemic thromboembolic complications, or other adverse events.

## RESUMO

**Introdução:**

o ácido tranexâmico (AT) ganhou reconhecimento em diversas especialidades cirúrgicas na prevenção sangramentos e complicações associadas, porém seu uso na cirurgia plástica ainda é limitado. O objetivo deste estudo foi avaliar a eficácia e segurança do uso tópico do AT na cirurgia reparadora oncológica da face e escalpo.

**Metodologia:**

foi realizado um ensaio clínico randomizado, duplo-cego, em pacientes maiores de 18 anos, portadores de neoplasias malignas de pele na região da cabeça (CID-10 C44.9). Os desfechos avaliados foram volume de perda sanguínea no pós-operatório imediato, hemorragia transoperatória de difícil controle, hematomas, equimoses, isquemia, necrose de tecidos, infecção de ferida operatória, efeitos tromboembólicos e outras intercorrências.

**Resultados:**

foram incluídos 54 pacientes, 26 no grupo AT e 28 no grupo placebo. O sangramento médio foi de 11,42ml, DP 6,40 (8,83 a 14,01) no grupo AT e de 17,6ml, DP 6,22 (15,19 a 20,01) no grupo controle, representando uma diminuição média de 6,18ml (35,11%) nas perdas sanguíneas (p=0,001). O AT reduziu significativemente o risco de equimoses (grupo AT: 1/26, 3,9% vs. grupo placebo: 23/28, 82,1%; p=0,000), representando uma redução relativa de 95,4% (RR=0,046; IC de 95%: 0,007-0,323). Houve dois casos de isquemia cutânea e um caso de necrose no grupo placebo, sem outras complicações no grupo AT.

**Conclusão:**

o uso tópico do AT foi efetivo na redução do sangramento transoperatório e pós-operatório imediato, com importante diminuição no risco de equimoses, não sendo evidenciados sofrimento isquêmico de retalhos, complicações sistêmicas tromboembólicas ou outros eventos adversos.

## INTRODUCTION

Tranexamic acid (TA) is an antifibrinolytic drug that blocks, by competition and with high affinity, the lysine binding sites on plasminogen molecules and inhibits plasmin formation, preventing fibrinolysis and clot dissolution^1^ ^,^ ^2^. TA has recently become an important subject of discussion, with a large number of randomized controlled trials^3^ ^,^ ^4^ and meta-analyses^5^ ^,^ ^6^ showing a reduction in blood loss, hemorrhagic events, and subsequent transfusions of blood products, especially in the areas of cardiac and orthopedic surgery^7^. Currently, more research in several surgical areas focuses on the transoperative topical administration of TA, to the detriment of intravascular administration, to obtain better results and fewer adverse effects^8^ ^,^ ^9^. There are still few references in the literature regarding the use of TA in plastic surgery, especially on its topical use in reconstructive surgery in the head region (face and scalp). Topical TA can be an auxiliary therapeutic modality in these procedures, preventing complications, reducing bleeding in the intraoperative and immediate postoperative periods, avoiding hematomas, ecchymosis, or other possible complications, such as tissue ischemia, necrosis, infections, and thromboembolic effects of intravenous use^10^ ^,^ ^11^. The aim of this study is to evaluate the intraoperative use of TA and its outcomes in reconstructive plastic surgery of the head.

## METHODS

### Study design and location

**Figure 1:**
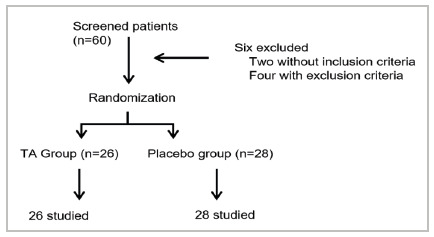
Flowchart of patient selection.

We conducted a randomized, double-blind, parallel-group, placebo-controlled clinical trial (Figure 1) at a university hospital in Southern Brazil between June 2021 and March 2022. This project followed the guidelines of Resolution No. 466, of December 12, 2012, and was submitted to the Teaching and Research Management and to the Ethics in Research Committee of the institution, under opinion number 4,899,188. A detailed analysis of the protocol is available at https://ppgcs.furg.br. All patients signed an informed consent form. This study was also conducted and registered under the Consolidated Standards of Reporting Trails (CONSORT guidelines) under number U1111-1290-7077.

### Inclusion and exclusion criteria

We included adult patients (18 years of age or older) with skin and/or deep tissue malignant lesions in the head region, including the face or scalp, classified by the ICD10 44.9.

We excluded patients with a body mass index greater than 35 kg/m^2^, smokers, patients with a score greater than or equal to 3 according to the American Society of Anesthesiologists (ASA) classification, users of anticoagulant medications or platelet inhibitors, patients with a history of thromboembolic disease and/or blood dyscrasias, and those with a report of side effects or allergy to TA.

### Intervention and control

We randomized participants into two groups: intervention and control. The former consisted of patients who underwent topical transoperative application of TA solution. The solution was distributed in a transparent vial, and the surgical site was irrigated during surgery with 10 ml of this solution. The remaining 15 ml was used in a pad that remained at the surgical site for 2 to 3 minutes. 

The control group was submitted to placebo, which consisted of the distribution of exclusively 25 ml of 0.9% saline, without TA, in a transparent vial, administered in the same way during the surgery. The surgeons did not know which solution they were applying, nor the patients. The randomization list was generated using the online computer program random.org. Blinding to random allocation was performed using identical sequentially numbered containers containing TA or placebo.

### Variables and definitions

The main outcome was the volume of blood loss in the immediate postoperative period. The secondary outcomes were: 1) difficult-to-control intraoperative hemorrhage; 2) postoperative hematoma; 3) postoperative ecchymosis; 4) ischemic processes at the surgical site; 5) necrosis at the surgical site; 6) surgical site infections; and 7) thromboembolic effects: deep vein thrombosis (DVT) or pulmonary thromboembolism (PTE).

The independent variables were: 1) group allocation: topical intraoperative use of TA or placebo; 2) sociodemographic data: age, sex, marital status, classified as single, with a partner, separated/divorced and widowed, skin color, classified according to the criteria of the Brazilian Institute of Geography and Statistics (IBGE): white, black, brown, asian, and indigenous, and categorized as brown, black, and white; 3) characteristics of the lesion or disease: clinical diagnosis of the lesion (performed by anamnesis and physical examination by two independent surgeons; histopathological diagnosis of the lesion (by histopathological study of the specimen removed during surgery); topography of the lesions in the head region (scalp, frontal, periorbital, nasal, malar, upper labial, lower lip, mental, submental/cervical, auricular, preauricular, and retroauricular), lesion size, classified as continuous measurement in millimeters based on its two dimensions of width and length; presence of systemic neoplastic disease, presence of skin lesion at birth, classification of surgical treatment, resection with primary closure; grafting; local retail; use of tissue expanders; number of surgical procedures required.

### Procedures and data collection

The indication of surgical procedures was performed by two surgeons. In case of disagreement, a third evaluation was arranged to reach a consensus. The procedures were performed in the operating room. Postoperative evaluations of patients were performed in the hospital wards during the hospitalization period and during consultations at the hospital outpatient clinic after discharge. During the procedure, the opening of the envelope with the indication (TA or placebo) and the delivery of the solutions (TA or placebo) to the surgeon were performed by previously trained medical school students, and the surgeon was not aware of whether he actually applied the TA medication or the placebo: since the solutions were colorless and in the same volume and the vials were the same, the solutions looked identical. 

To evaluate the outcomes, we applied anamnesis and serial physical examinations, with the application of specific questionnaires developed for the study, reporting complications and specific characteristics of the patients. The first questionnaire was applied one week before the procedure and completed on the day of the procedure immediately after surgery. The second questionnaire was applied and filled in the immediate postoperative period, with data being complemented according to the postoperative evaluations on the following days up to the seventh day, with revision at 21 and 28 days. 

### Statistical analysis

For data processing and analysis, we built a database in the Epidata 3.1 program. Two independent typists double-entered the data. Subsequently, the database was cleaned to identify amplitude or consistency errors, and the database was translated into the Stata 13.1 statistical program. Initially, we performed a descriptive analysis of the sample. We used the chi-square or Fisher’s test to compare the dichotomous data between the intervention group and the placebo group. For continuous data, we used the Student’s t-test to compare the two groups. In all analyses, we considered a p-value lower than 0.05 for a two-tailed test.

## RESULTS

We included 54 patients in this trial, 26 in the TA Group and 28 patients in the placebo group (Figure 1). All patients had malignant skin neoplasms located in the head region (face or scalp) and none had systemic neoplasia. Histologically, 39 patients had basal cell carcinoma (72.22%), 14 had squamous cell carcinoma (25.92%), and one had melanoma (1.85%). 

There was no significant difference in the distribution of baseline demographic characteristics or in relation to the size and location of malignancies between the intervention and placebo groups (Table 1).

**Table 1:** Baseline data of patients and their lesions (n=54).

Data	TA group (n = 26)	Placebo group (n=28)	p
Age (years, mean ± SD)	60.65 ± 15.65	63.82 ± 14.27	0,44
Skin color (n/%)			
Brown	0 / 0	2 / 7.1	1.0
White	26 / 100	26 / 92.9	
Sex (n/%)			
Female	18 / 69.2	16 / 57.1	0.3
Male	8 / 30.8	12 / 42.9	
Lesion size (cm, mean ± SD)	1.73 ± 0.75	1.72 ± 0.59	0,95
Topography of lesions (n/%)			
Scalp	2 / 7.69	4 / 14.28	0,67
Frontal	2 / 7.69	4 / 14.28	0,67
Periorbital	4 / 15.38	1 / 3.57	0,18
Nasal	5 / 19.23	5 / 17.85	1,0
Malar	8 / 30.76	3 / 10.7	0,09
Upper lip	2 / 7.69	3 / 10.7	1,0
Submentonian/cervical	0 / 0	2 / 7.14	0,49
Auricular	1 / 3.08	1 / 3.57	1,0
Pre-auricular	2 / 7.69	5 / 17.85	0,42

Regarding the type of reconstructive surgical procedure, in the TA group there were surgical flaps in 76.92% of the patients, primary closure of lesions in 15.38%, and skin grafts in 7.69%; in the placebo group, surgical flaps were used in 60.71% of the patients, primary closure in 25%, and skin grafting in 14.28%. There was no significant difference in the distribution of treatment types between groups.

The mean bleeding amount in the immediate postoperative period in the TA group was 11.42ml (SD 6.40, range 8.83-14.01), and in the placebo group it was 17.6ml (SD 6.22, range 15.19-20.01). The TA group showed an average decrease of 6.18ml in blood loss (35.11%) when compared with the placebo group (p=0.001) (Table 2).

**Table 2:** Outcomes in the TA and placebo groups (n=54).

Variable	TA group (n = 26)	Placebo group (n=28)	p
Bleeding ml (mean ± SD)	11.42 ± 6.40	17.60 ± 6.22	0.001
Hematoma (n/%)	0 / 0	2 / 7.1	1.0
Ecchymosis (n/%)	1 / 3.8	23 / 82.1	0.000
Ischemia (n/%)	0 / 0	2 / 7.1	1.0
Necrosis (n/%)	0 / 0	1 / 3.6	1.0
Surgical site infection (n/%)	0 / 0	0 / 0	-
DVT (n/%)	0 / 0	0 / 0	-
PTE (n/%)	0 / 0	0 / 0	-
Reintervention (n/%)	0 / 0	1	1.0
Other effects (n/%)	0 / 0	0	-
Satisfaction (average)	10	9	1.0

Regarding hematoma, two patients in the placebo group had this complication in the postoperative period and none in the TA group, but without statistically significant difference (Table 2).

Ecchymosis was a common complication in patients in the placebo group, occurring in 23 patients (82.1%), and in only one (3.8%) in the TA group (Table 2), with a relative risk (RR) of 0.046 (95% CI 0.007-0.323). 

Only two patients belonging to the placebo group presented ischemia in flaps performed in reconstructive surgical procedures, and in one of them there was a need for surgical reintervention due to flap necrosis in a nasal reconstruction. There were no cases of surgical wound infection, nor were there any thromboembolic phenomena, such as DVT or PTE, or any other adverse event caused by the topical use of TA in any of the studied groups (Table 2).

As for the degree of patient satisfaction with the results obtained in the procedures, there was no significant difference between the two groups studied. All patients had a level of satisfaction with a mean higher than nine in both groups, on a scale of 1 to 10.

## DISCUSSION

The present study is the first randomized clinical trial conducted to evaluate the efficacy and safety of the topical intraoperative use of TA in reconstructive plastic surgery in the face and scalp region for oncological lesions. According to Wang’s metanalysis, topical intraoperative application of TA in orthopedic surgeries may limit systemic absorption and decrease the risk of thromboembolic complications, while providing a higher therapeutic concentration and better induction of microvascular hemostasis^10^. The preparation and concentration of the drug were based on previous studies on its topical use in orthopedic surgery, obtaining a 3% solution, which corresponds to the dilution of three vials of TA 250mg/5ml in 10ml of 0.9% saline, obtaining 750mg of the substance in 25ml of solution^12^.

In the evaluation of blood loss in the immediate postoperative period, we used gravimetry, i.e., the measurement of the weight of blood in the material used, such as pads and gauze, with subsequent deduction of the dry weight of the items. The calculations were performed individually for each surgical procedure, using a previously calibrated analytical balance. Weight in milligrams (mg) was converted to volume for the estimation of blood loss in ml, considering the mean density of human blood as approximately 1.060g/ml^13^. Our study showed a reduction in mean intraoperative and immediate postoperative bleeding, with a mean decrease of 6.18ml in blood loss (35.11%) in patients undergoing topical intraoperative use of TA (p=0.001). It was also characterized by the differential of having used gravimetry to calculate blood losses, which we believe provides a better estimate of intraoperative and immediate postoperative losses than, for example, hematocrit measurements because, according to what has been reported in the literature, the evaluation of hematometric indices such as hemoglobin (Hb) or hematocrit (Ht) can be erroneous: in patients with acute hemorrhage, postural hypotension may occur in 36 to 48 hours due to volume loss, but Hb and Ht levels will still be normal until they undergo hemodilution as the patient is rehydrated^14^ ^-^ ^16^.

Regarding the evaluation of possible secondary outcomes, for the definition of difficult-to-control intraoperative hemorrhage, was considered it the excessive bleeding resistant to surgical maneuvers, which would hinder the performance of the procedure and lead to blood loss above 15% of blood volume^17^ ^,^ ^18^; postoperative hematoma was defined as transient lesions due to large collections of blood pigments in the interstitium, to the point of causing local bulging and compression of adjacent structures^19^, with no difference between the groups. The absence of hemorrhages and hematomas in the TA group, although not statistically significant, is a favorable result that stimulates research with novel studies and larger samples. On the other hand, as for postoperative ecchymosis, which would be a transient lesion with a violet color due to the presence of blood pigment, in which small portions of red blood cells infiltrate the interstitium^20^, we observed a relative reduction of 95.4% (RR=0.046; 95% CI 0.007-0.323) in patients undergoing intraoperative topical use of TA, demonstrating a statistically significant difference and its importance in prevention. 

Ischemic processes at the surgical site were sought by clinical observation of coloration, capillary return, edema, and temperature of flaps and grafts^21^; necrosis at the surgical site was characterized by pale and cold darkened skin, causing loss of reconstruction, either by primary closures, grafts, or flaps^22^; and surgical site infections, by clinical evaluation of purulent incision drainage, pain, increased sensitivity, swelling, hyperemia or warmth in tissues, deep spontaneous scar dehiscence, fever (temperature ≥38ºC) and/or positive culture of secretion or tissue from the superficial incision, obtained aseptically, within the first 30 days after the surgical procedure^21^ ^,^ ^22^. Ischemic processes, necrosis and surgical wound infections did not occur in the group submitted to TA application, demonstrating that the drug did not have negative effects on the viability of surgical flaps, not leading to ischemic processes in this type of procedure, in which vascularization impairment is a possible complication. Regarding systemic complications, the TA group also did not present thromboembolic effects, including deep vein thrombosis (DVT) or pulmonary thromboembolism (PTE), with clinical diagnosis performed according to the guidelines and algorithms of the European Society of Cardiology (ESC) and the Wells scores for DVT or PTE^23^ ^,^ ^24^, or any other reported side effect, which corroborates findings that topical use of the medication may decrease or nullify this risk^25^.

Among five studies identified in the literature review with a specific approach to the use of TA in plastic surgery, in only two studies TA was administered topically, and in the other intravenous use was performed. All these studies, in diverse types of plastic surgery procedures, corroborate our findings regarding the reduction of blood loss and the absence of complications, in particular thromboembolic phenomena^20^ ^,^ ^26^ ^-^ ^29^. 

Regarding the patient’s own assessment of the degree of satisfaction with the treatment result, there was no significant difference between the two groups. The high averages of satisfaction degree in both groups correlate with previous studies in reconstructive plastic surgery and quality of life, emphasizing the characteristics and psychological profile of the patient who resorts to reconstructive surgery, with focus and expectation on improving the function and treatment of the disease, a key factor capable of generating good results^30^.

In addition to the reduction of surgical risk for the patient, there is also a reduction in costs, since the topical use of TA requires a lower dose than the intravenous one, thus being a more economical and effective approach. 

## CONCLUSION

Topical intraoperative use of TA in oncological reconstructive plastic surgeries in the head (face and scalp) region decreased intraoperative and immediate postoperative bleeding, reducing ecchymosis and not leading to complications such as ischemic processes of flaps and grafts, necrosis or infections, or systemic complications such as DVT and PTE, among others. Further studies are needed to explore the influence of patient characteristics, surgical site, type of procedure, and the influence of treatment on outcomes.
